# Innovative concept for a major breakthrough in atmospheric radioactive xenon detection for nuclear explosion monitoring

**DOI:** 10.1007/s10967-013-2525-8

**Published:** 2013-05-17

**Authors:** G. Le Petit, A. Cagniant, M. Morelle, P. Gross, P. Achim, G. Douysset, T. Taffary, C. Moulin

**Affiliations:** 1 CEA, DAM, DIF, 91297 Arpajon, France; 2 Canberra Semiconductor NV, Olen, Belgium

**Keywords:** Radioactive xenon, Beta/conversion electron-X-ray/photon coincidence, SPALAX™, Silicon detector, CTBT

## Abstract

The verification regime of the comprehensive test ban treaty (CTBT) is based on a network of three different waveform technologies together with global monitoring of aerosols and noble gas in order to detect, locate and identify a nuclear weapon explosion down to 1 kt TNT equivalent. In case of a low intensity underground or underwater nuclear explosion, it appears that only radioactive gases, especially the noble gas which are difficult to contain, will allow identification of weak yield nuclear tests. Four radioactive xenon isotopes, ^131m^Xe, ^133m^Xe, ^133^Xe and ^135^Xe, are sufficiently produced in fission reactions and exhibit suitable half-lives and radiation emissions to be detected in atmosphere at low level far away from the release site. Four different monitoring CTBT systems, ARIX, ARSA, SAUNA, and SPALAX™ have been developed in order to sample and to measure them with high sensitivity. The latest developed by the French Atomic Energy Commission (CEA) is likely to be drastically improved in detection sensitivity (especially for the metastable isotopes) through a higher sampling rate, when equipped with a new conversion electron (CE)/X-ray coincidence spectrometer. This new spectrometer is based on two combined detectors, both exhibiting very low radioactive background: a well-type NaI(Tl) detector for photon detection surrounding a gas cell equipped with two large passivated implanted planar silicon chips for electron detection. It is characterized by a low electron energy threshold and a much better energy resolution for the CE than those usually measured with the existing CTBT equipments. Furthermore, the compact geometry of the spectrometer provides high efficiency for X-ray and for CE associated to the decay modes of the four relevant radioxenons. The paper focus on the design of this new spectrometer and presents spectroscopic performances of a prototype based on recent results achieved from both radioactive xenon standards and air sample measurements. Major improvements in detection sensitivity have been reached and quantified, especially for metastable radioactive isotopes ^131m^Xe and ^133m^Xe with a gain in minimum detectable activity (about 2 × 10^−3^ Bq) relative to current CTBT SPALAX™ system (air sampling frequency normalized to 8 h) of about 70 and 30 respectively.

## Introduction

Since the opening for signature of the Comprehensive Test Ban Treaty (CTBT) in New York on September 24th 1996 with the goal to ban nuclear test explosions in any environment, the verification regime in place over years through the International Monitoring System (IMS) is today nearly reaching its nominal effectiveness relying on three different wave form technologies (seismic, infrasound and hydro-acoustic) and two radionuclide technologies (atmospheric aerosols and noble gas). In case of low yield underwater or underground nuclear explosion, it is admitted that only radioactive gases, especially noble gas difficult to contain (nearly inert gas), can be released to the atmosphere through dynamic venting or atmospheric pumping [1–4]. Among them four radioactive isotopes of xenon, ^131m^Xe, ^133m^Xe, ^133^Xe and ^135^Xe produced in fission processes and exhibiting suitable half-lives and radiation emissions can be detected in atmosphere far away from the site release. Their detections provide an undisputable evidence of a nuclear test relative to the wave form technologies that could bring only presumption of an illicit test in case of low nuclear yield. This was typically the case regarding the nuclear test conducted by Democratic People’s Republic of Korea (DPRK) in 2006 reported by a Swedish noble gas system similar to an IMS one [5]. On May 25th 2009, DPRK announced that it had conducted a second nuclear test and waveform signals recorded for this event at seismic stations around the globe exhibit characteristic of a nuclear explosion [6], however no radioactive xenon emissions correlated to the event was measured by the IMS network. This case implies, if the 2009 DPRK test is a nuclear one, that the explosion gases were quite well contained in the cavity of the nuclear experiment or either the xenon emission releases were too weak to be detected by the noble gas station network, in spite of their high sensitivity, or not distinguishable from the global radioactive xenon background originating from nuclear power plant operations and from medical isotope production plants [7–12]. Identify a specific radioactive xenon isotope signal present in air, far away from the expected release site, as the one emanating from a nuclear weapon explosion is not trivial even using isotopic ratio analysis, which can be very complex to exploit, since many source and scenario factors play a key role in the associated signature as presented in several studies [13, 14].

Better sensitivity is therefore still needed for radioxenon monitoring systems in order to enhance the detection of a prohibited nuclear experiment. Furthermore, such improvement will allow for higher sampling frequency limiting the impact of atmospheric dilution (as well as influence of civilian source terms) and therefore, together with atmospheric transport modelling, improving the test site localisation. Several ways to reach this goal have been summarized in a recent work [15], such as larger air volume sampled using new dry compressors with a better xenon extraction and improvements in purification/concentration processes with the use of new membranes/adsorbents or new technologies, improvement in detection efficiencies and time measurement by coupling several detectors, drastic reduction of the detector overall background and above all by considering a complete refinement of the radioxenon monitoring sub-system detection. This paper focus on a new efficient based-silicon detector concept for beta and conversion electron (CE) emission measurement coupled to a well-type NaI(Tl) for X-ray and gamma radioxenon emission measurement, both detector exhibiting a very low radioactive background. This detector combination leads to a new compact spectrometer allowing CE/β-X/γ coincidence detection with high efficiencies and a powerful isotope discrimination due to a high electron energy resolution.

## Importance of the radioxenon system’s sample time frequency

The four systems developed for the CTBT verification regime, i.e. the Swedish Automatic Unit for Noble gas Acquisition [16], the automated radioxenon sampler and analyser (ARSA, Bowyer et al. [17] 1996), the Analyser of Xenon RadioIsotopes (ARIX, Y. [18]), the Système de Prélèvement Automatique en Ligne avec l’Analyse du Xénon atmosphérique (SPALAX™, [19]), perform radioxenon measurement either using high-resolution HPGe gamma spectroscopy (SPALAX) or β–γ coincidence spectroscopy from a plastic scintillator coupled with a NaI(Tl) detector (ARSA, ARIX and SAUNA). Whatever the system, minimum detectable concentrations (MDCs) for isotopes ^133^Xe and ^135^Xe are quite similar (respectively ≈0.1 and 0.5 mBq.m^−3^) but lower for the metastable isotopes with the β–γ systems due to better emission probability of the decay modes considered in this technique. However, owing to their poor energy resolutions, especially for the electron signals (≈30–35 %) [20], these systems aren’t taking advantage of their better sensitivity for the isotopes ^131m^Xe and ^133m^Xe since many interferences (or memory effect) in the coincidence region of interest (ROI) have to be solved, involving sophisticated algorithms [21, 22]. In the other hand, the SPALAX™ system exhibits a longer air sampling frequency (24 h compared to 12 h for the other systems) which can be a drawback for the closest noble gas stations owing to the effect of the atmospheric dilution of any original signal during the transport of the contaminated air masses.

**Fig. 1:**
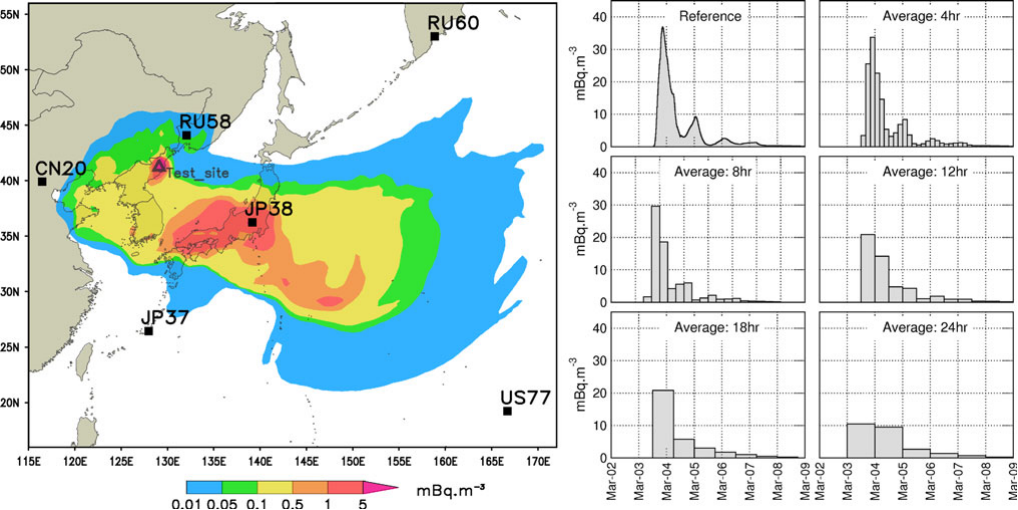
Average activity concentrations, expressed in mBq.m^−3^, matching a virtual release from the DPRK site in the time range T0–T0 + 8 days (*left part*, *open triangle*) and simulated activity concentrations calculated over time at the JPX38 IMS site (Takasaki-Japan, *black square*) by varying the noble gas station sampling time from real meteorological wind fields (*right part*)

In order to improve sensitivity of the SPALAX™ IMS noble gas station in atmospheric radioactive xenon detection from a new spectrometer design and consequently to allow better event discrimination [23], one should define and focus on some key parameters. One of them which will directly impact the detector features is related to the sampling frequency. One aims to reach is weighting up the pros and con between the potential gain in detection and the associated complexity of the station operational cycle. Figure 1 illustrates the influence of the average sampling time on the calculated activity concentration levels from a hypothetical release case. In this example, a simulated release from the DPRK test site (open triangle) involving a 10^13^ Bq source term is continuously emitted during 1 h. The dispersion of the plume is then calculated from real meteorological wind field over 8 days from the initial event occurrence (T0, initiated on March 1, 2012). The left part of the Fig. 1 shows the average activity concentrations, expressed in mBq.m^−3^, in the time range T0–T0 + 8 days, while the right part exhibits the activity concentrations calculated over time at the JPX38 IMS site (Takasaki, Japan) by varying the virtual noble gas station sampling time. This example clearly shows that a sampling time less than 12 h leads to a stronger detected signal, close to a factor up to 3 compared to a 24 h sampling period systems. Although the case studied here do not encompass all the different situations likely to occur, depending on both the release phenomenology and the meteorological conditions prevailing at time of the event, it appears that a 8 h sampling time (in coherence with the ^135^Xe half-life) is a good compromise likely to lead to a substantial gain in the amplitude of the signal to be detected without additional complexity of the operational IMS system’s gas sample processing for keeping an high detection sensitivity.

## Key parameters for high electron detection performances

Table 1 gives the most important decay modes related to the four relevant radioactive xenon isotopes (^131m^Xe, ^133m^Xe, ^133^Xe and ^135^Xe) underlining that X-ray-CE coincidence counting is the most efficient way for measuring the two metastable isotopes, ^131m^Xe and ^133m^Xe. However, their conversion electron energies, 129 and 199 keV respectively are too closed to one another to be distinguished (unless spectra are measured over time) with the current IMS β–γ systems that use a plastic scintillation cell for high electron efficiency but exhibit poor energy resolution, close to 30 % at 200 keV [24]. Moreover, the coincidence between the 81 keV gamma line of ^133^Xe and its preceding beta emission on the one hand and the coincidence between the 249 keV gamma line of ^135^Xe and its preceding beta emission on the other hand, are likely to lead to strong interferences in the ^131m^Xe and ^133m^Xe electron spectra area when gated by the 30 keV X-ray line since the Xe and Cs X-rays cannot be separated with the NaI(Tl) detector.

**Table 1:** Main decay modes for the four relevant radioactive isotopes of xenon with associated emission probabilities in bracket [25]

Isotope	^131m^Xe	^133m^Xe	^133^Xe	^135^Xe
Half-life (days)	11.84	2.20	5.24	0.38
Major γ-ray energy (keV)	163.9 (1.98 %)	233.2 (10.3 %)	81.0 (37 %)	249.8 (90 %)
K X-ray energy (keV)	30.4 (54 %)	30.4 (56.3 %)	31.7 (47.6 %)	31.7 (5.2 %)
β Endpoint energy (keV)			346 (99 %)	905 (96 %)
Conversion electron energy (keV)	129.0 (61.0 %)	198.7 (63.5 %)	45 (53.0 %)	214 (5.7 %)

In order to avoid these drawbacks, a new technology drastically improving the electron energy resolution has to be coupled to the NaI(Tl) detector which has the advantages of exhibiting a high photon detection efficiency, especially for X-ray at 30 keV, as well as allowing to operate at ambient temperature ensuring ease of use, especially for remote stations. A silicon detector based on the PIN diodes technology appears to be the most suitable for beta and electron measurement since it shows an excellent electron energy resolution (3.3 % at 45 keV), low electron energy threshold (close to few keV) and a proper efficiency till several hundred of keV, depending of the thickness of the silicon wafer used. However, if these features are currently achieved for small surface detector (<30 mm^2^ [26, 27], it is very challenging for a room temperature very large PIN diode (larger than 1,000 mm^2^) to achieve high performances in energy resolution (<10 %) and in low electron energy threshold (<40 keV), suitable for ^131m^Xe and ^133m^Xe CE detection, Indeed, such areas are mandatory for improving the SPALAX detection limits matching to an 8 h sampling frequency and delivering a final processed air volume of about 10 cm^3^ (purified and concentrated xenon in a nitrogen media).

This apparent quest for the Holy Grail results from the need to independently optimize several key linked parameters while each of them evolves in antagonistic ways leading to worsen the global performances. This is illustrated by one of the key parameters, i.e. minimization of the electronic noise of the silicon detector achieved by reducing its total capacitance. Hence, the larger silicon depletion depth the lower the detector capacitance and higher the leakage current are, which involves in return to degrade both electron energy resolution and energy threshold. The average silicon pathway for electron at 346 keV (^133^Xe β endpoint) is about 300 μm. As a result, in order to optimize the detection of the ^135^Xe β emission (Table 1) without drastically altering the electron energy resolution, it is more appropriate to target a maximum silicon thickness depletion of about 500 μm. Once this key parameter settled, the leakage current should be minimized, especially considering the large silicon ship area (1,232 mm^2^) required for optimizing the electron efficiencies. In order to reach the best performances for both electron energy resolution and energy threshold, stringent selection of the silicon chips for proper specifications matching high spectroscopy performances has been performed. Consequently, all the selected large silicon wafers exhibit very low leakage current, <20 nA, and allow an excellent electron full width at half maximum close to 10 % at 200 keV while energy threshold obtained is around 30 keV, which is greatly suitable for the detection of the lower conversion electron at 45 keV (^133^Xe). From these key physical features CEA and Canberra Semiconductor company have jointly designed a new electron detector, hereafter described, exhibiting a low radio-impurities contamination and high performance in detection sensitivity.

## New SPALAX™ radioxenon electron photon detection design

**Fig. 2:**
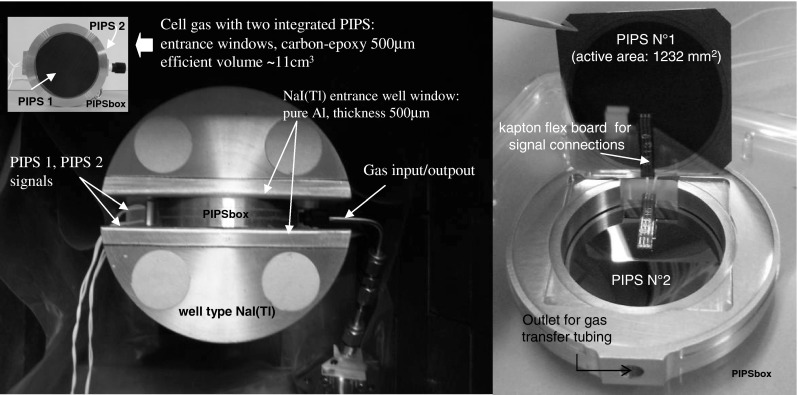
New conversion electron–photon coincidence spectrometer developed by CEA/Canberra Semiconductor. View of the electron detector equipped with two passivated implanted planar silicon (PIPSbox) surrounded by a well type low background NaI(Tl) for photon detection (*left*), internal details of the PIPSbox (*right*)

The detection system has been designed for operating at room temperature and in order to be coupled with a SPALAX™ gas sample process providing automatically injection and removal of gaseous xenon sample through a thin pipe. The detection system consists in a gas cell with two large passivated implanted planar silicon (PIPS, based on PIN diode technology) surrounded by a well-type NaI(Tl) crystal (Fig. 2).

**Fig. 3:**
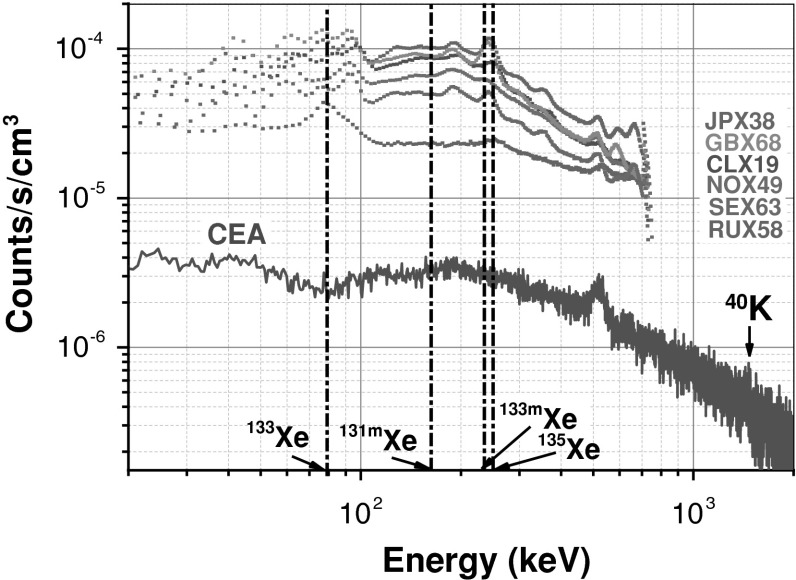
NaI(Tl) background measurement comparison between several detectors that equipped the β–γ noble gas stations of the CTBT IMS network (SAUNA stations JPX38, GBX68, CLX19, NOX49, SEX63 and ARIX station RUX58) and the new detector design

The NaI(Tl) size (*ϕ* 102 × 76 mm height), has been designed considering background reduction by minimizing photon detector volume (511 cm^3^) while allowing a complete energy deposition at 250 keV (^135^Xe) together with maximizing the detection solid angle. The window enclosing the well crystal and facing the gas cell is constituted by a thick (500 μm) pure aluminium layer, minimizing the X-ray attenuation at 30 keV. Specific care in selecting the photomultiplier tube was carried out in order to drastically reduce ^40^K impurities in the glass window components that are likely to provide significant gamma background from Compton interaction processes. Figure 3 presents the comparison between the NaI(Tl) background related to the CEA design with those currently measured for some equipments of the CTBT International Monitoring System (SAUNA and ARIX noble gas stations). As it can be observed, a gain in background reduction of at least one decade, over the Xe isotopes photon energy range (Table 1), is obtained with this new set-up.

By the same token and in order to keep the background as low as possible for the whole electron-photon spectrometer, all materials composing the gas cell equipped with the PIPS (hereafter called PIPSbox) have been selected with regard to their radioactive purity and/or minimizing as low as possible their mass. As the silicon chips used for manufacturing the PIPS detectors exhibit high purity despite their large areas, the associated radioactive background is expected to be negligible. The component with the higher mass is the aluminium housing the two face-to-face PIPS™ with only 58 g owing to a sizeable active gas volume of 11 cm^3^. However, this PIPSbox component is not the highest contributor to the background since low background aluminium is used. Hence, the three components with the expected largest activity are the carbon epoxy PIPSbox window, despite of a low thickness (500 μm) fitted for X-ray transmission at 30 keV higher than 95 %, the tubing dedicated to gas inlet and finally the epoxy glue used for the carbon window sealing and the PIPS™ wires for detector signals read out. The epoxy used for sealing and gluing some PIPSbox internal components is the same used by Canberra Semiconductor manufacturer for low background germanium detectors. Screening tests have led to a value of 3.5 mBq g^−1^ for ^238^U, which is low considering the mass used in the detector. For the electric circuit, a Kapton^TM^ flex board was used for its advantage of being very thin and for its low radioactive background [28]. As connectors are possible source of noise, four long wires coming out of the PIPSbox and welded to LEMO™ connectors are used for extracting signals of the two PIPS™. Due to the complex assembly process, each PIPS™ detector has its own ground contact with the aluminium housing box. Furthermore in order to minimize possible microphonic effects, the carbon epoxy window is aluminized and sealed vacuum-tight with a conductive epoxy to be at the same potential as the ohmic back side of the PIPSbox.

**Fig. 4:**
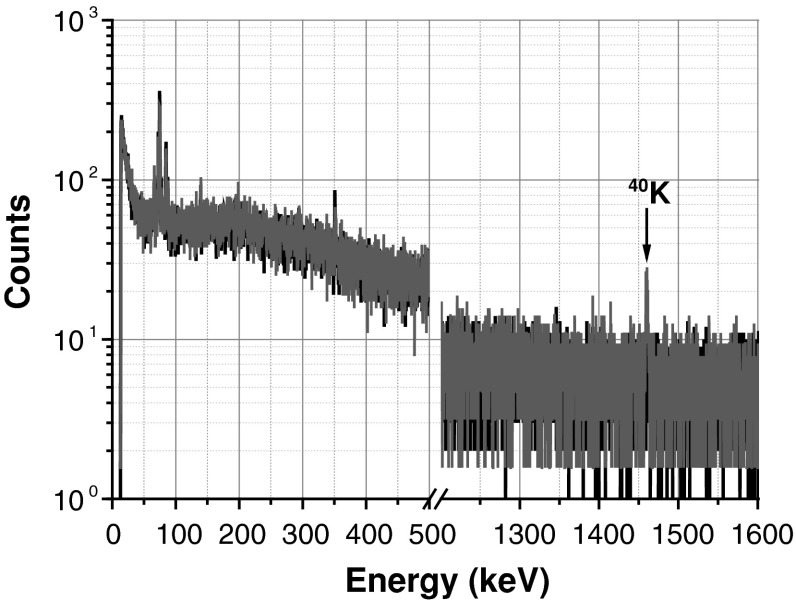
Germanium detector background measurement (*black curve*) and background spectrum (*blue curve*) of the PIPSbox placed on the top of the germanium detector. Both spectra normalized to the same acquisition time (150,000 s). The only gamma line detected is related to traces of ^40^K. (Color figure online)

As a result of this stringent detector set-up, Fig. 4 shows the resulting radioactive background of the electron detector (PIPSbox) estimated from a gamma emission spectrum using a low background germanium detector. The germanium background spectrum (black) and the PIPSbox spectrum (blue) normalized to the same acquisition time (150,000 s) are quite perfectly overlapping, except at 1,460 keV corresponding to ^40^K traces. This extremely weak radioactive background related to the electron detector is of great importance for coincidence detection, especially for measurement of the metastable radioxenon (^131m^Xe and ^133m^Xe) at very low level from a 30 keV X-ray gated electron spectrum, since the background in the regions of interest (ROIs) is expected to be very closed to zero.

## CE and photon efficiency, CE resolution and threshold

The intrinsic electron detection efficiency of the PIPSbox detector is linked to the solid angle subtended by the two coaxial circular and parallel PIPS™ (radius *R*_s_ = *R*_D_ = 39.6 mm, distance *h* = 3.6 mm). The solid angle can be precisely evaluated from the following converging formula used in primary standardisation laboratories [29]:

$$ \frac{\Upomega }{4\pi } = 2 \times \frac{{R_{\text{D}} }}{{R_{\text{S}} }} \times \frac{1}{2n}\sum\limits_{i = 1}^{n} {\frac{{\sin^{2} \varphi }}{{\sqrt {x - \cos \varphi } \times \left( {\sqrt y + \sqrt {x - \cos \varphi } } \right)}}} $$

in which $x = \frac{{R_{\text{S}}^{2} + R_{\text{D}}^{2} + h^{2} }}{{2R_{\text{S}} R_{\text{D}} }}$, $y = \frac{{h^{2} }}{{2R_{\text{S}} R_{\text{D}} }}$ and *φ* = (*i* − 0.5) *π*/*n* and n is an integer value of choice.

The solid angle (*Ω*/4*π*) obtained is 0.526 ± 0.041. However, a lower experimental electron detection efficiency has been determined mainly due to PIPSbox dead volume and in a lesser extent to losses induced by specific physical phenomena such as backscattering electron effects at the PIPS™ surface or between the two PIPS™ (crosstalk) as well as absorption in the gas volume (consisting in 30 % Xe and 70 % N_2_ under a 1 bar pressure at standard conditions), although only significant for CE of ^133^Xe at 45 keV. The electron detection efficiencies have been determined using radioactive gaseous xenon standards (quasi pure ^131m^Xe, mixture of ^133^Xe and ^133m^Xe) delivered by the Radiation Seibersdorf laboratories (Austria). Within energy range from 45 to 199 keV, the average electron detection efficiency has been found to be 44 ± 5 % complying with the intrinsic efficiency previously determined from the angle solid calculation (52.6 ± 8 %).

The X-ray and γ detection efficiencies for gaseous sample corresponding to the spectrometer set-up previously presented (Fig. 2), have been determined with the same standards used for the electron detection efficiency calculation. The photon detection efficiency reaches 41 ± 5 % and 75 ± 9 % respectively at 30 and 164 keV (^131m^Xe) and 60 ± 7 % at 81 keV (^133^Xe). These values are comparable to those related to the existing CTBT β–γ systems.

**Fig. 5:**
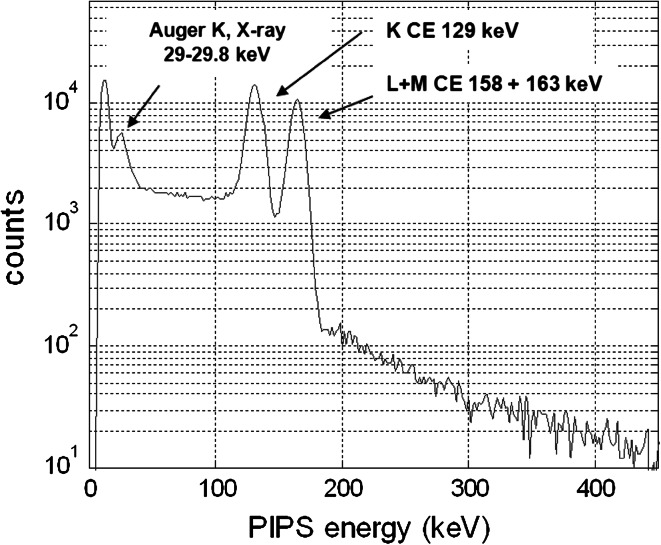
Direct conversion electron of ^131m^Xe measured from the PIPSbox detector filled by an almost pure radioactive gaseous standard (15.3 Bq). Auger electron at 29 keV and X-rays close to 30 keV are also present. Energy calibration was performed using ^131m^Xe CE energies and Αuger electron at 30 keV (acquisition time 57,955 s). Counts under 20 keV are caused by electronic noise

Figure 5 presents the ^131m^Xe conversion electron response of the PIPSbox detector filled with a pure gas standard (activity: 15.3 Bq). At 129 keV conversion electron peak is very well separated from the doublet corresponding to CE at 158 and 163 keV, with probability emission of 29 and 6.7 %, respectively due to a very good energy resolution (electron full width at half maximum around 9 keV at 129 keV). The lowest electron energy likely to be detected is about 29 keV as shown Fig. 5 where Auger electron at 29 keV (probability emission: 7 %) convoluted with X-ray close to 30 keV are observable. This low electron threshold allows detection of all K CE of the four xenon relevant radioactive isotopes without any energy overlap (Table 1). For three of them (^131m^Xe, ^133m^Xe and ^133^Xe), both their electron emission probability and detection efficiencies are larger than the corresponding photon detection features. Combined to a very low spectrometer background, CE-X-ray coincidence detection will therefore lead to a drastic improvement in detection sensitivity for both ^131m^Xe and ^133m^Xe and a very significant one for ^133^Xe. Considering this last isotope since electron at 346 keV (β end-point) is fully deposited in silicon, the β emission signal can be considered as enhancing the detection efficiency as discussed later. Regarding the isotope ^135^Xe, as the thickness depletion of silicon detector was optimized to 500 μm, only a part of its β spectrum will be fully detected (a thickness close to 1,250 μm would have been necessary in order to detect the β end-point energy at 905 keV). However, from the β spectrum shape it can be determined that the average β emission of ^135^Xe is about 300 keV [30], a value smaller to those corresponding to the maximum possible full energy absorption in the 500 μm silicon chip, estimated at 400 keV for a normal incidence direction and straight pathway. In fact owing to the geometry of the PIPSbox the average electron pathway is larger than the normal incident one, this means that at least 60 % of the β emission is fully detected (lower part of the spectrum). For the higher part of the spectrum the fraction of the electron energy deposed in the silicon is unknown. But this is not a drawback because one is thinking of quantifying the ^135^Xe by analysing the gamma spectrum gated by the full electron spectrum. In that case, it only needs an electron signal whatever the energy fraction deposed in the detector. With this approach it is expected to get an efficient detection sensitivity for ^135^Xe regarding both the gamma (250 keV) probability emission (90 %) and the beta probability emission (96 %) involved in this coincidence mode analysis.

## Electronics and multi-parameter data acquisition system

Signals from both NaI(Tl) and PIPSbox detector’s preamplifier are shaped with a CAEN N968 spectroscopy amplifier and digitalized with a FAST ComTec 7070 analog to digital converter (ADC). Each ADC signal is recorded with a multi-parameter data acquisition system from a FAST ComTec (MPA-3) unit. This MPA-3 has 4 inputs and is connected to a computer by a high–speed bus (PCI card). The MPANT software, used for signal acquisition and for spectra plotting, is recording the data from the MPA-3. This setup allows to simultaneously performing direct, coincidences and anti-coincidences measurements.

Each ADC used for electron and photon energy conversion can trigger an event by opening a coincidence window. For the radioxenons of interest, electron and photons are emitted within less than 10 ns (the largest time difference between coincidental emission of an electron and a photon related to radioxenon decays is for emission following the beta decay of ^133^Xe to an excited state of ^133^Cs, this state having a 6.36 ns half-live time). Signal shaping time used are 6 μs for photons and 2 μs for electronic signals (sufficient to collect the complete charge) in order to reduce dead time of the electronic acquisition system while maximizing energy resolution. Based on these features, a coincidence window time set to 10 μs was used to record data leading to an appropriate compromise between signal loss and signal gain (random coincidences). For each analog signal larger than the ADC internal threshold (20 mV), the ADC sends a BUSY signal to the MPA-3. This BUSY signal triggers a counter waiting during the coincidence time window for BUSY signals from any ADCs. If two BUSY signals from two different ADCs are recorded during the coincidence time window, then the corresponding signals will be considered as coincident (or anti-coincident depending on the specification) and stored in a three dimensions spectrum. Specifications can be set, as channel range for one of the ADCs signal in order to provide additional gated coincidence spectra.

## CE-X-ray coincidence detection performance for ^131m^Xe

In order to have better statistics and consequently a precise view of the 3D spectra, high radioxenon activity concentration compared to the usual environmental ones have been measured for demonstrating the performances of the new detection system shielded by 5 cm thick bricks of low activity lead.

**Fig. 6:**
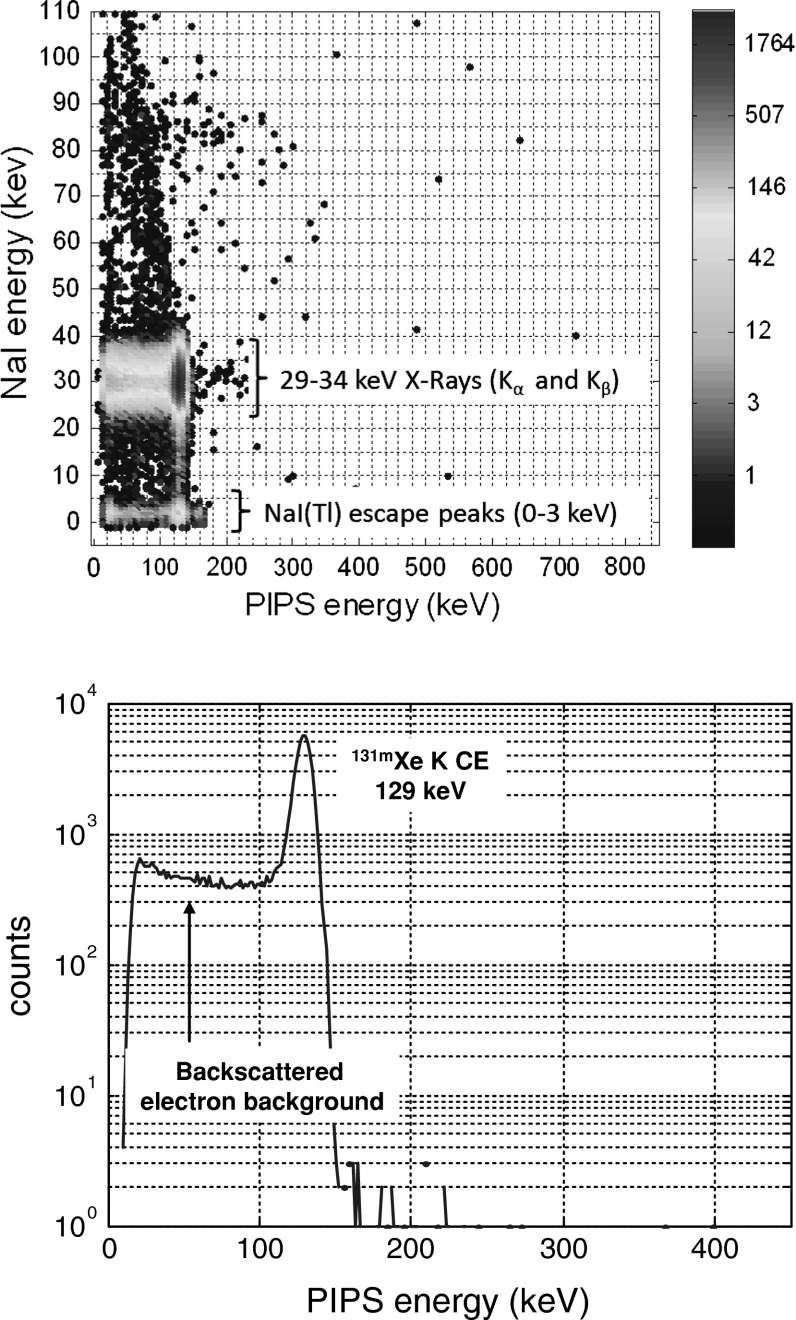
3D electron-photon spectrum of ^131m^Xe (*upper part*) and 129 keV K CE peak of ^131m^Xe gated by 30 ± 12 keV X-ray area (*lower part*) achieved from a 14.5 Bq spike measured by the well NaI(Tl)/PIPSbox spectrometer (acquisition time: 66,061 s)

Figure 6 (upper part) shows the 3D electron-photon spectrum of a 14.5 Bq pure spike of ^131m^Xe injected in the PIPSbox. The most intense area is related to coincidence detection between X-rays close to 30–34 keV measured by NaI(Tl) and electrons measured by the PIPSbox, the red plot corresponding to coincidences with full deposit of ^131m^Xe 129 keV K shell CE. The least intense area is related to coincidence events between ^131m^Xe 129 keV K shell CE and Kα, Kβ xenon X-rays, these coincident events are placed on the 2 keV photon energy line because the corresponding xenon X-ray has undergone an iodine X-ray escape. The iodine X-ray escape happen when a low energy photon from the sample undergoes a photoelectric interaction with a iodine atom located near the surface of the NaI(Tl) crystal. There is then a chance that the X-ray produced by the reorganization of the targeted atom escapes the crystal. For a photon of energy hν (hν being close to the sensitive material K X-ray), the total energy deposited in the detector of a photon undergoing an iodine K X-ray escape is hν-E_K X-ray_. In the present case, xenon K X-ray undergoing an iodine K X-ray escape will deposit only 2 keV in the sensitive part of the detector.

Figure 6 (lower part) shows the gated X-ray (30 ± 12 keV) 129 keV K shell CE spectrum. It should be noted that 158 and 163 keV L shell CE (present in the ungated spectrum of Fig. 5) are not visible in the spectrum since they are in coincidence with 3.7 and 5.3 keV X-ray lower than NaI(Tl) energy threshold. The electron continuum towards lower energy from the 129 keV CE peak is caused by variable energy losses of electron due to interaction at the silicon chip surface and backscatter effects.

The 129 keV K CE peak from the gated spectrum (Fig. 6, lower part) has been fitted with a Gaussian function, that is a proper choice taken into account the global uncertainty budget, including the one related to the counting volume cell, on the ^131m^Xe activity standard (14.5 ± 1.0 Bq 1*σ*, at the time of measurement). The associated peak efficiency for the selected coincident events has been found to be 92 ± 13 count/s/kBq a much larger value compared to a recent one recently published for ^131m^Xe [31] and by the past.

**Fig. 7:**
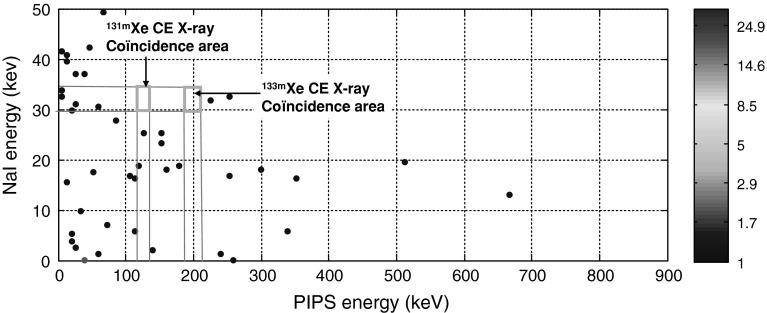
3D coincidence background spectrum related to the NaI(Tl)/PIPSbox system shielded by 5 cm of lead, acquired on 240,000 s with the well NaI(Tl)/PIPSbox detection system. No count were detected in both ^131m^Xe and ^133m^Xe region of interests (ROIs)

In order to assess a possible memory effect of the PIPSbox likely to impact the following measurement, the PIPSbox has been removed after each experiment and connected to a bench gas transfer where it has been kept under a secondary vacuum during 10 h in order to remove the whole gas volume for eliminating all radioactive xenon traces. Figure 7 shows the coincidence background spectrum acquired using this procedure after the ^131m^Xe spike measurement presented (Fig. 6). Although the process used is not complying with those currently use by IMS noble gas stations (for instance the SPALAX™ counting cell undergoes a vacuum of only 30 min each 24 h cycle processing), the result is very promising since not only no memory effect has been observed but also the background is drastically low, especially the ROIs matching the expected coincidence events related to ^131m^Xe and ^133m^Xe. Hence, none count in the ^131m^Xe and ^133m^Xe ROIs has been measured after more than 2 days measurement, as shown in the 3D coincidence background spectrum (Fig. 7).

Disregarding decay factors during air collection, processing and measurement (in a current IMS sampling and measurement cycle, the global correction factor for ^131m^Xe accounts for <10 %), the sensitivity of β–γ detector systems is expressed as minimum detectable activity (MDA) using the simplified following formula [16, 32, 33], given for a confidence level of 95 %: ${\text{MDA}} = \frac{2.71 + 4.65 \times \sqrt B }{{t \times \varepsilon_{\gamma } \times \varepsilon_{e} \times P_{e} \times P_{\gamma } }}$, where *B* is the coincident background for the considered event area (*B* is determined from the X-ray gated electron spectrum), *t* is the acquisition time, *ε*_*γ*_, *ε*_*e*_, *P*_*γ*_, *P*_*e*_ are respectively the photon and electron detection efficiencies and their associated emission probabilities. As the NaI(Tl)/PIPSbox systems background is closed to zero and as no memory effect contributes to the background, the 4.65$\sqrt B$ term is negligible compared to 2.71. Consequently, for a given acquisition time, the only parameters likely to improve the MDA are both a gain in the electron and photon detection efficiencies. MDAs calculated for ^131m^Xe and ^133m^Xe from a coincidence background spectrum of the well NaI(Tl)/PIPSbox system (cell filled with air under NTP condition) have been calculated to be (2.0 ± 0.3)0.10^−3^ Bq for 1 day of measurement. Compared to the MDA’s achieved from current detection system currently integrated in the CTBT SPALAX systems, the gain using the new system is respectively of about 70 and 30 which is a major improvement.

Considering a CTBT SPALAX™ noble gas system operating with a 8 h sampling time resolution and equipped with the new detection system, minimum detectable activity concentrations (MDCs) calculated for ^131m^Xe and estimated for ^133m^Xe have been found to be (1.8 ± 0.3).10^−4^ Bq.m^−3^ (1 day measurement basis). Compared to the MDC currently achieved by an IMS SPALAX™ system (air sampling frequency normalized to 8 h), improvement in detection sensitivities for the two metastable isotopes are respectively of a factor 30 and 10. These lower gains in MDC compared to the MDA ones are due to the fact that the final gas volume processed remains equal to 25 cm^3^ whatever the sampling time resolution used by the system and consequently do not comply with the PIPSbox active volume. In order to better take advantage of the new detection system performances, it will be appropriate to reduce the nitrogen media, used for flushing the gas xenon during the concentration and purification steps of the SPALAX process, present in majority in the sample (70 % of the whole amount) for fulfilling the 11 cm^3^ of the PIPbox cell with a sample more enriched in xenon. In return the MDCs will be greatly further enhanced (studies of such modification are under progress).

## CE-X-ray coincidence detection performance for a mixed ^131m^Xe, ^133^Xe environmental sample

**Fig. 8:**
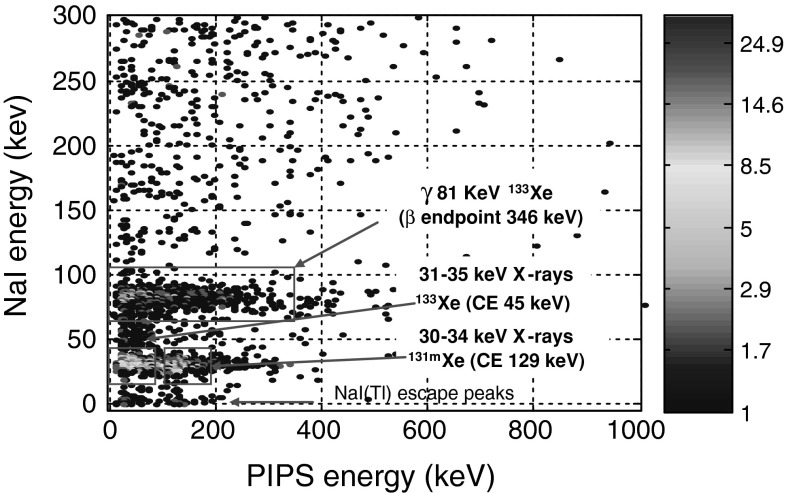
3D electron-photon spectrum of ^133^Xe and ^131m^Xe acquired from a re-analysis of an archive bottle related to an environmental sample processed by a CTBT type noble gas station located in Ottawa (Canada). Acquisition time: 68,234 s

In order to evaluate spectrometry performances of the new detection system in case of a mixed xenon radioactive isotopes, the PIPSbox cell has been filled using a bench gas transfer with an air environmental sample from an archive bottle processed by a CTBT type noble gas station located in Ottawa (Canada). The activity of ^133^Xe and ^131m^Xe contained in the sample were respectively 180 ± 16 and 163 ± 15 mBq (the isotopic ratio corresponds to the signature of the Chalk River isotope production facility releases). Figure 8 shows the NaI(Tl)/PIPSbox 3D coincidence spectrum obtained with a 68,234 s acquisition time.

The former radioactive isotope ^133^Xe is detected from coincidence events of the 81 keV photon with 0–346 keV β emission along with the converted 81 keV γ transition leading to 45 keV CE in coincidence with the β continuum. The later (^131m^Xe) is detected from coincidence events between Kα, Kβ X-rays and K shell CE at 129 keV as already observed in the previous chapter.

**Fig. 9:**
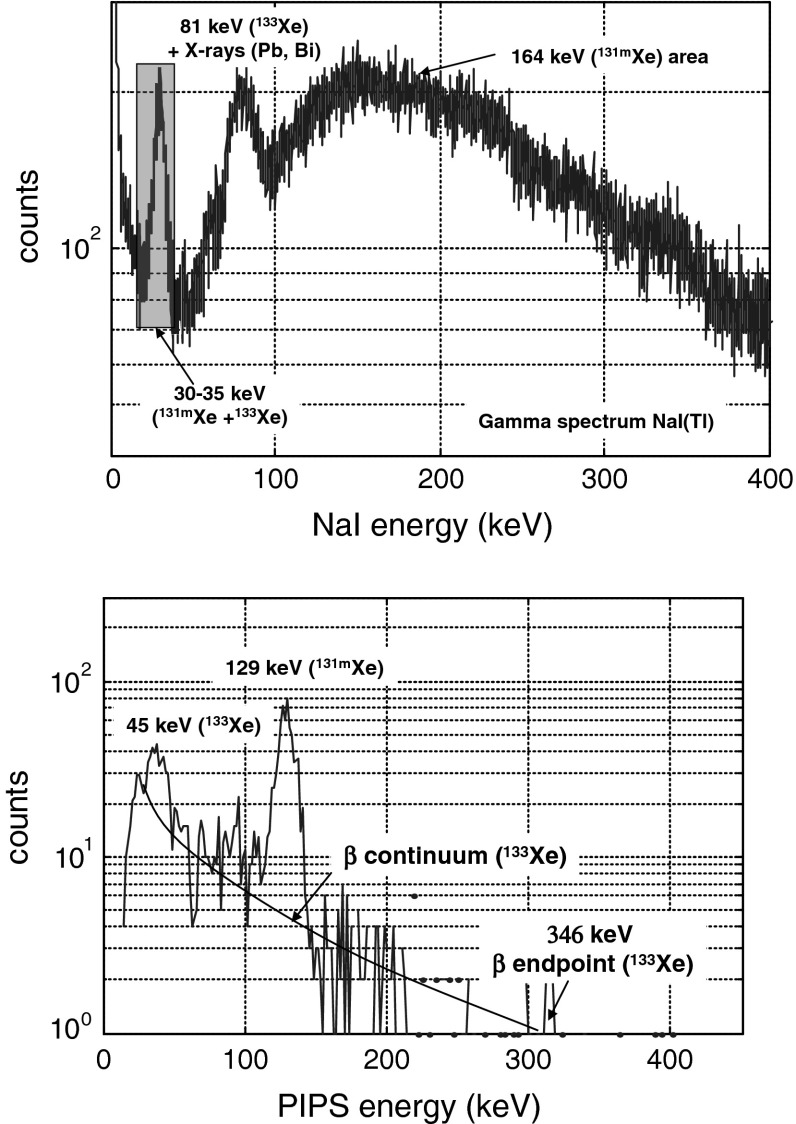
Direct NaI(Tl) photon spectrum (*upper*) and X-ray gated electron PIPSbox spectrum (*lower*) related to a re-analysis of an archive bottle related to an environmental processed air sample by a CTBT type noble gas station located in Ottawa (Canada). The electron spectrum, gated by X-ray (*purple* energy window) exhibits the 45 keV and 129 keV K shell CE peaks respectively related to ^131m^Xe and ^133^Xe. The CE peaks are superimposed to the β emission continuum related to ^133^Xe (endpoint energy at 346 keV). Acquisition time: 68,234 s. (Color figure online)

Figure 9 presents the direct NaI(Tl) X and gamma spectrum (upper part, ^131m^Xe gamma line at 164 keV is not detected) and the gated electron spectrum (lower part) by the X-ray energy window defined on the photon emission spectrum (upper part). The ^133^Xe K shell CE peak at 45 keV is clearly detected with a slight alteration of its energy resolution owing to higher predominance of energy straggling at low energy following loss of energy of electrons in the N_2_/Xe gas mixture. From the combination of the β emission and K shell CE measured from the X-ray gated electron spectrum, it is expected to achieve for ^133^Xe, a MDC comparable to those determined for ^131m^Xe by using a list-mode data analysis and specific offline (or even on line) post-analysis as the signals from detector preamplifiers can be digitalized and recorded event by event with amplitude and timestamp. Hence, these results underline the dependence of the ^131m^Xe MDC as a function of the ^133^Xe activity concentration, in case of a mixed sample, due to the contribution of the ^133^Xe β continuum under the conversion electron peak at 129 keV. Future works will deal with these needed complementary studies and analysis developments.

## Conclusion

It has been successfully demonstrated that a new detection spectrometer using innovative technologies can improve key requirements for detection radioxenon monitoring (CTBT expresses a MDC <1 mBq.m^−3^ for ^133^Xe on a 1 day measurement basis) and could be worthwhile integrated in the near future, in the frame of CTBT, to existing noble gas stations for improving detection sensitivity while allowing higher sampling frequency.

The compact CE-photon spectrometer conceived by CEA in collaboration with Canberra Semiconductor company combines a gas cell with two integrated large silicon chips for detecting electrons and a well-type NaI(Tl) embedding the cell and specifically designed for full absorption of photons up to 250 keV (higher relevant photon energy related to the radioxenon decays). The silicon technology (PIPS™) used allows large areas (1,200 mm^2^) while keeping a very low electron energy threshold (about 30 keV) thanks to a stringent selection of the silicon chips. Consequently, PIPSbox cell geometry allows a quite important active gas volume to be measured, close to 11 cm^3^ (about half the gas volume processed in a current IMS SPALAX™). In return taking advantages over the existing noble gas systems to the excellent resolution for CE peak (<7 keV at K shell CE 129 keV), the very low coincidence background (no count measured during 2 days), the absence of any memory effect (in the tested operational conditions) and high efficiencies for both electrons and photons, the new detection system leads to a drastic reduction of minimum detectable activities (MDAs) for radioxenons compared to existing CTBT SPALAX™ detection system. First assessments of MDAs, determined from the coincidence background spectrum achieved from the NaI(Tl)/PIPSbox spectrometer, have led to MDAs of about 2 × 10^−3^ Bq (1 day measurement) for both metastable radioactive isotopes of xenon (^131m^Xe and ^133m^Xe), that is respectively about 70 and 30 times lower than those achieved previously. However, the MDCs that might be achieved by replacing the current detection system of the French noble gas system by the new one will be “only” improved by a factor of respectively 30 and 10 (8 h sampling time) since the current SPALAX™ gas sample processing method do not allow to modify the final gas volume. Consequently, works will aim at reducing the gas media (nitrogen) much below 70 % (current concentration in the sample) by modifying the SPALAX™ sample processing and thus taking advantage of the performances of the new spectrometer to lower further the MDCs. In parallel, works will be performed to achieve precise MDC’s for the other relevant radioxenons (^133^Xe, and ^135^Xe) taking advantage of a numeric data acquisition system (PIXIE-4) that will be coupled to the new detection system. With these new improvement, using list-mode specific data analysis, it can be expected to reach MDCs <1.10^−4^ Bq.m^−3^ (8 h sampling time) for the four relevant radioxenons.
